# Recent advances in pediatric bladder malignancies

**DOI:** 10.12688/f1000research.19396.1

**Published:** 2020-02-25

**Authors:** Roberto Iglesias Lopes, Marcos Figueiredo Mello, Armando J. Lorenzo

**Affiliations:** 1 Pediatric Urology Unit, Division of Urology, Hospital das Clínicas, University of São Paulo Medical School, São Paulo, 05402-000, Brazil; 2 Division of Urology, The Hospital for Sick Children and Department of Surgery, University of Toronto, Toronto, ON, Canada

**Keywords:** PEDIATRIC, BLADDER MALIGNANCIES

## Abstract

Urothelial pediatric neoplasms are relatively rare. Papillary urothelial neoplasms of low malignant potential (PUNLMPs) and rhabdomyosarcoma (RMS) are the most common bladder malignancies in the pediatric population. Clinical presentation encompasses macroscopic hematuria or lower urinary tract symptoms (or both) or is detected incidentally at imaging. Tumors arising from the bladder can originate from any of its four histological layers (urothelium, lamina propria, detrusor, and adventitia) and are divided into tumors that have an epithelial origin (arising from the urothelium) and those that have a non-epithelial origin (mesenchymal neoplasms). RMS is the most common malignant tumor of the urinary bladder in children younger than 10 years. Deriving from the embryonic mesenchymal cell, the histopathologic subtypes of RMS are embryonal RMS (>90%) and alveolar histology (<10%). Pre-treatment imaging should be carried out by computed tomography (CT) or at present is more likely with magnetic resonance imaging of the pelvis. Chest CT and bone scintigraphy are used to screen for metastases. In selected cases, a positron emission tomography scan may be recommended to evaluate suspicious lesions. The current prognostic classification considers age, histologic subtype, tumor site, size, and extent (nodal or distant metastases). Staging is based on pre-operative findings, group is based on intra-operative findings and pathology, and risk stratification is derived from both stage and group data. Pre-operative chemotherapy is the most common first-line intervention for bladder/prostate RMS, before surgery or radiation therapy. Collaborative groups such as the Soft Tissue Sarcoma Committee of the Children’s Oncology Group and the European Pediatric Soft Tissue Sarcoma Study Group endorse this therapy. PUNLMPs are generally solitary, small (1–2 cm), non-invasive lesions that do not metastasize. Therapy is usually limited to a transurethral resection of the bladder tumor. About 35% are recurrent and around 10% of them increase in size if they are not treated.

## Introduction

Bladder tumor occurrence and histological subtypes differ considerably between adults and children. Pediatric bladder neoplasms are relatively rare and—with the exception of rhabdomyosarcoma (RMS)—are associated with favorable benign behavior and outcomes after adequate therapy. The incidence does appear to be increasing, according to some studies such as data analyses between 1973 and 2003 using the Surveillance, Epidemiology and End Results (SEER) database. Papillary urothelial neoplasm of low malignant potential (PUNLMP) and RMS are the most common bladder malignancies in the pediatric population. Males were generally more affected than females with bladder malignancies (by a ratio of 2:1) ^1^.

Clinical presentation includes macroscopic hematuria or lower urinary tract symptoms or both (dysuria, frequent urination, incontinence, pelvic pain, and urinary retention) and a palpable suprapubic mass or is detected incidentally at imaging ^2^.

**Figure 1.**
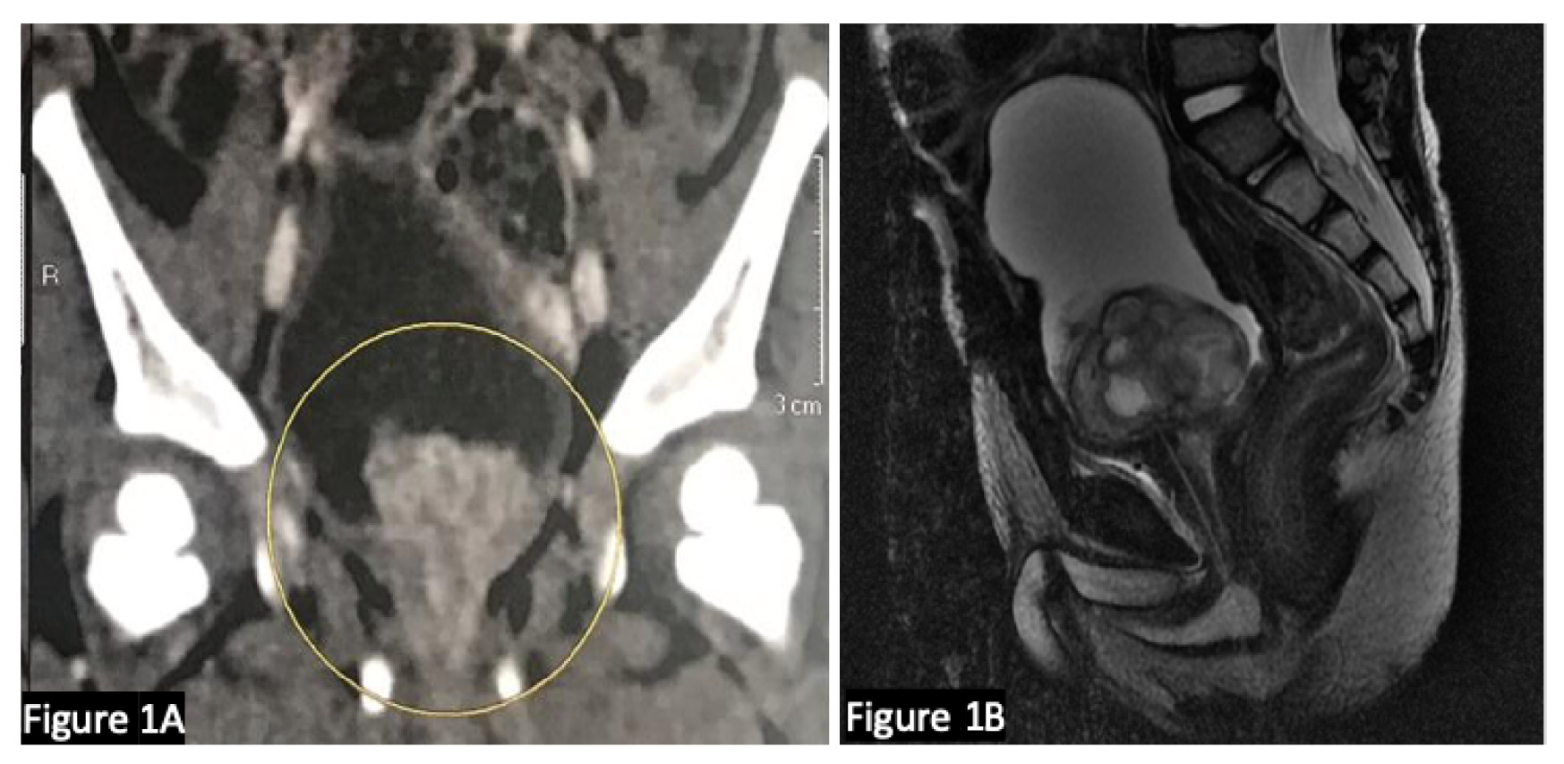
Computed tomography and magnetic resonance images of tumor. ( **A**) Computed tomography depicting bladder tumor at the trigone. ( **B**) Magnetic resonance image showing better characterization of location and extent of the tumor. We confirm that the patients gave us permission to use these images.

**Figure 2.**
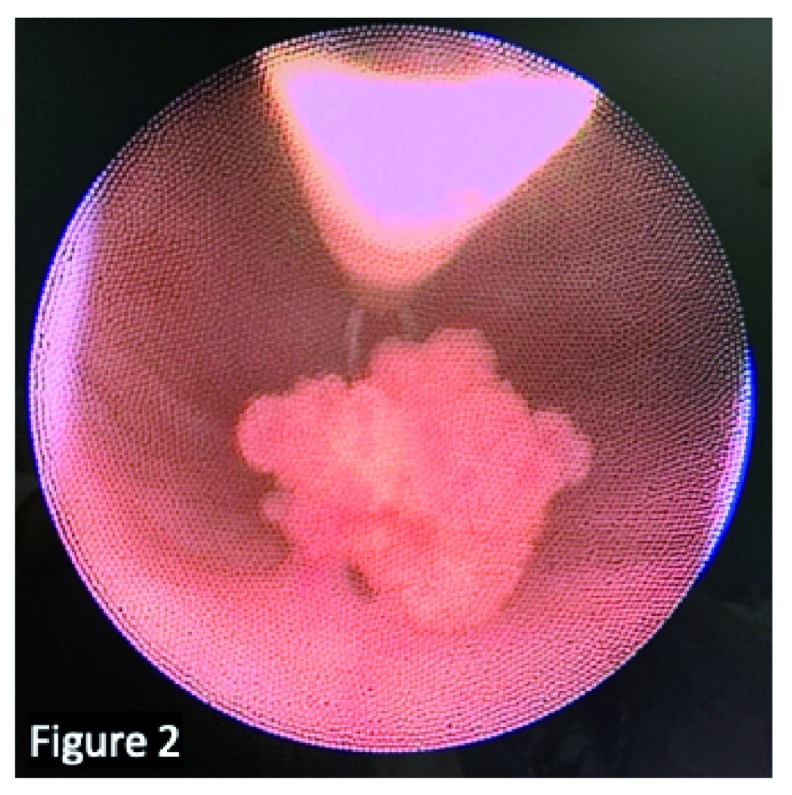
Transurethral resection of a solitary, non–muscle invasive, and low-grade papillary urothelial neoplasms of low malignant potential. We confirm that the patient gave us permission to use this image.

Urinalysis and urinary culture are mandatory to exclude or confirm concomitant urinary tract infection. Owing to the low sensitivity (low cell turnover related to the benign nature of many pediatric bladder lesions) and the absence of experience of pediatric pathologists in such situations, urinary cytology is rarely useful ^2^. Ultrasonography (US) is the most common initial examination: a full bladder during examination is advisable to avoid missing small lesions or making a misinterpretation ^3^. If malignancy is suspected, pelvic computed tomography (CT) or—preferably—magnetic resonance imaging (MRI) is performed for better characterization of the location and extent of disease ( Figure 1). A tissue biopsy is often necessary, usually through cystoscopic resection, since imaging alone is not able to predict histological subtypes ( Figure 2). Work-up includes hemoglobin quantification in case of massive hematuria, assessment of inflammatory marker levels if suspicion of infection and determining renal function if bladder tumor is associated with hydronephrosis.

## Subtypes

Tumors arising from the bladder can originate from any of its four layers (urothelium, lamina propria, detrusor, and adventitia) and are classified as tumors with an epithelial origin (urothelial tumors) or a non-epithelial origin (mesenchymal neoplasms) ^4^. Urothelial neoplasms are rare in children. Non-invasive urothelial neoplasms are much more common in children than are invasive urothelial carcinomas ^5^. Other relevant but rarer tumors in the pediatric population will also be discussed in this article.

### Mesenchymal neoplasms

RMSs are malignant tumors deriving from the embryonic mesenchymal cell, which forms the striated musculature. In the genitourinary tract, RMS could affect the bladder, prostate, paratesticular region, uterus, and vagina. RMS is the third most common extracranial solid tumor of childhood (after Wilms’ tumor and neuroblastoma). Overall, 15 to 20% of all RMSs arise from the genitourinary tract. RMS is also the most common malignant tumor of the urinary bladder in children younger than 10 years ^1^, accounting for 5% of all childhood solid cancers ^6^. Males are more commonly affected than females with bladder malignancies (by a ratio of 2:1) ^1^. We assume that, in boys, genitourinary pelvic RMS originates mainly on the prostate and more rarely on the bladder, but owing to the initial extent of the tumor, RMS appears as a bladder prostate primary tumor in the literature. In females, genitourinary RMS originates mostly on the bladder but can rarely be primary from the vagina or uterus.

Genitourinary pelvic RMSs have a bimodal age distribution; the peak incidence occurs within the first 2 years of life (>50% of cases, mostly embryonal or botryoid RMS) and another peak occurs during adolescence (mostly alveolar RMS) ^6^. Age of diagnosis is an important risk factor for genitourinary pelvic RMS: rates of event-free survival (EFS) are 53% for those who are less than 1 year or more than 10 years old and 71% for those who are 1 to 9 years old ^7^. Sporadic tumors are much more frequent (90% of all cases), but association with genetic syndromes should be considered (Li–Fraumeni, neurofibromatosis type 1, multiple endocrine neoplasia type 2A, and *DICER1* germline pathologic variant, especially in pelvic female RMS) ^8, 9^.

**Figure 3.**
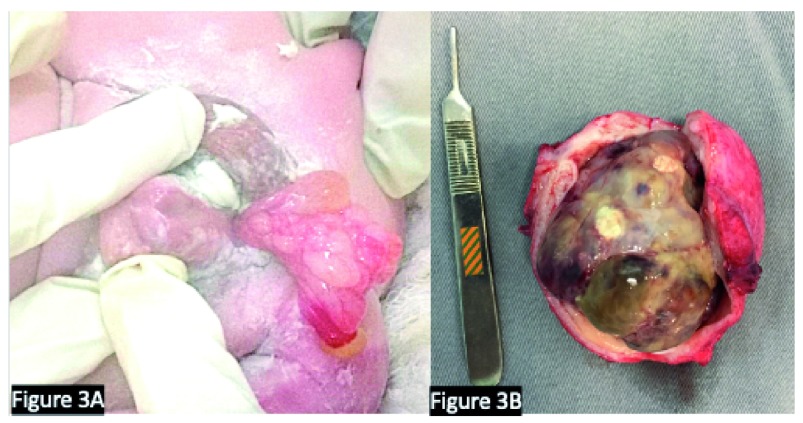
Cases of rhabdomyosarcoma. ( **A**) A male infant at 14 months with congenital rhabdomyosarcoma protruding through the urethra. Note the association with a proximal hypospadias. ( **B**) Cystoprostatectomy specimen showing extensive rhabdomyosarcoma with necrosis after neoadjuvant chemotherapy. (Surgery was performed for the tumor depicted in frame **B**.) We confirm that the patients gave us permission to use these images.

The histopathologic subtypes of RMS include embryonal RMS (>70–90% of cases), are more common in children younger than 10 years of age, and have a favorable prognosis. The alveolar histology (<10–30% of cases) is more frequent in adolescents and confers a lower chance of cure. Macroscopically, these lesions are typically polypoid and gelatinous when they occur in cavities and multilobulated when they are of the botryoid subtype, as shown in Figure 3. Microscopically, the embryonal subtype consists of small, dark, spindle-shaped, or round cells with minimal cytoplasm, mixed with a variable number of cells resembling rhabdomyoblasts. The alveolar subtype is characterized by thin septae lined by a single layer of cuboidal tumor cells with hyperchromatic nuclei resembling alveolar airspaces ^10^.

Pre-treatment imaging preferably should be carried out by CT or MRI of the pelvis and abdomen for nodal area evaluation. The chest should be evaluated by CT. Bone scintigraphy is useful for screening for osseous metastases. Positron emission tomography CT (PET-CT) scan is progressively being used instead in the evaluation of this neoplasm. Bone marrow biopsies are also needed.

After initial work-up, extirpative surgery is indicated for patients whose tumors are localized and amenable to complete resection with minimal morbidity, which is really uncommon in bladder primary neoplasms: only about 12% are likely to be amenable to complete excision at presentation ^10^. Pelvic and retroperitoneal nodes at the renal artery or below can be affected. Bladder function can be saved in up to 60% of cases by partial cystectomy: while dome tumors are easily resected, lower or extensive lesions require reimplantation of ureters or bladder augmentation (or both) or a neobladder ^11– 13^.

Stratification of the risk of RMS is based on a pre-treatment TNM (tumor–lymph nodes–metastasis) staging system and a post-biopsy and resection clinical grouping system established by the Intergroup Rhabdomyosarcoma Study Group (IRS-G), now contained within the Children’s Oncology Group (COG) Soft Tissue Sarcoma Committee. Through the performance of several multicenter randomized trials, this Committee classified the RMS more accurately by tailoring treatment to optimize cancer outcome and minimize treatment-related effects. The current challenge is the risk stratification of tumors by biological characteristics to provide targeted therapies ^14, 15^.

The COG classification is divided into pre-treatment staging and clinical groups ( Table 1). Pre-treatment staging is based on size, site, and extent of the tumor (nodal or distant metastases), and clinical groups are divided on pre- and intra-operative findings. Risk groups take all of these data into account along with pathology and biologic behavior analysis ( Table 2). Localized embryonal RMS has a better prognosis with an 80% 5-year EFS rate, whereas the alveolar subtype is associated with a 65% 5-year EFS rate ^14, 15^.

**Table 1.** Intergroup Rhabdomyosarcoma Study clinical groups and pre-treatment staging (bladder only).

Pre-treatment staging		Clinical groups	
1	None of bladder tumors fall in this category Only genitourinary sites (other than bladder and/or prostate)	I	Completely resected, no evidence of metastatic disease
2	≤5 cm, negative lymph nodes and no metastases	II	IIA. Microscopic residual disease after complete gross resection IIB. Positive lymph nodes with no residual disease IIC. Positive lymph node with microscopic residual disease
3	≤5 cm, positive lymph nodes or >5 cm with any nodal status, no metastases	III	Gross residual disease
4	Metastatic disease	IV	Distant metastases

**Table 2.** Current Children’s Oncology Group-Soft Tissue Committee risk stratification for rhabdomyosarcoma (bladder only).

Risk group	Pre-treatment stage	Clinical group	Histology
Low	2	I–II	Embryonal
	3	I–II	Embryonal
Intermediate	2–3	III	Embryonal
	2–3	I–III	Alveolar
High	4	IV	Embryonal
	4	IV	Alveolar

Quality of the surgery has a major impact on local disease control and correlates with the prognosis. IRS clinical groups are used for disease classification and prognosis ( Table 1). European Pediatric Soft Tissue Sarcoma Study Group (EpSSG) stratification is composed of four risk groups: (1) low risk: small tumors (<5 cm), completely resected at diagnosis (IRS group I) with favorable histology (embryonal, botryoid); (2) intermediate risk: resected at diagnosis with microscopic residual disease (IRS group II) with favorable histology (embryonal, botryoid); (3) high risk: large IRS group III tumors (>5 cm) with or without nodal involvement or alveolar subtype or both; (4) very high risk: metastatic disease (IRS group IV).

Collaborative groups (COG and EpSSG) offer multimodal treatment—local therapy (radiotherapy or surgery) and chemotherapy—in an attempt to improve outcome whilst finding the correct balance between the intensity of treatment and its possible cost in terms of late effects.

If tumor resection is not possible (for instance, when excision with clear margins will cause functional impairment or mutilation), a diagnostic biopsy is required.

Group I: completely resected disease and can be treated with surgery only or by additional post-operative chemotherapy. Chemotherapy based on vincristine, actinomycin, cyclophosphamide (VAC) or ifosfamide, vincristine, actinomycin (IVA) combinations is the most common first-line intervention in COG. The EpSSG protocol advised only for vincristine, actinomycin (VA) regimen to reduce long-term gonadal toxicity of alkylating agents.

Group II: resected tumor with microscopic residual disease. Need of further treatment is unclear in this scenario. Nevertheless, as for all RMSs, adjuvant chemotherapy is usually required, and radiation therapy is often applied.

Group III: gross residual disease after resection, which is the most common situation. Usually, it presents with an unsatisfactory prognosis, and adjuvant local treatment after chemotherapy is a point of debate and study; EpSSG places more emphasis on surgery, and COG on radiation therapy.

Radiation therapy is frequently needed and commonly employed. In general, this is employed for the following cases: (1) unresectable residual tumor, (2) residual disease following surgery, (3) nodal disease, and (4) alveolar histology. There is considerable debate regarding what the best strategy for local control should be. With the ultimate goal of organ preservation, surgery and radiation therapy offer different risks and benefits. Although radiation often allows for lower initial morbidity and preservation of the affected organ, the long-term effects from radiation remain worrisome, especially in young boys (median age of this disease is 2 years old) ^16^. On the other hand, surgery often requires aggressive resection and reconstruction that relies on bladder substitution and need for catheterization. Furthermore, the implications of radiation after surgery and surgery after radiation are debatable, and both generate important concerns in patients who fail the primary local control modality. These issues will persist until data from multi-institutional groups mature. Brachytherapy is a good alternative to apply radiation therapy on the pelvis and is gaining traction in some parts of the world. As a general principle, a conservative strategy must be preferred with partial cystectomy and radiotherapy, rather than radical cystectomy. As long-term effects with brachytherapy appear to be reduced in comparison with external radiotherapy, this technique must be considered, especially in young patients. Proton therapy may replace brachytherapy if the latter technique is not available.

Group IV: metastatic disease that presents a poor prognosis with an overall survival rate of 20 to 30% and whose management has not improved over time; therefore, experimental approaches are needed for these patients ^17^.

Surveillance imaging (for at least 5 years) is recommended for patients. The disease recurs in up to a third of the patients, and 95% of relapses occur within 3 years ^12, 13^.

### Inflammatory myofibroblastic bladder tumors

Inflammatory myofibroblastic tumors of the bladder (IMTBs) are rare (a quarter of these neoplasms occur in children) and are characterized by a benign and reactive proliferation of myofibroblasts. A review of 42 reported cases of pediatric IMTB showed equal prevalence in males and females. Clinical presentation includes hematuria, dysuria, or abdominal pain, and mean age at presentation is 7.5 years (range of 2 to 15 years). The etiology of IMTB is poorly understood and is attributed to infectious or traumatic causes or a possible clonal lesion mainly involving the anaplastic lymphoma kinase gene ( *ALK-1*) or *NTRK*, *ROS1 PDGFR*, and *NTRK*, which are far more common in children and young adults ^16^.

Lesions range in size from 1.8 to 13 cm, and the mean size is 5.5 cm in the largest diameter. Lesions may be pedunculated, nodular, lobular, or frond-like in appearance. The commonest sites of IMTBs are the dome of the bladder in children and the lateral bladder wall in adults. Tissue biopsy is essential for diagnosis. Immunohistochemistry ALK-1 expression is useful in the diagnosis of IMTB but is not always present ^16^. Nowadays, systematic RNA sequencing is needed to confirm this diagnosis.

Complete surgical resection of the lesion is the treatment of choice. In children, no proven recurrent/metastatic IMTB has been reported, but follow-up is warranted as recurrent IMTB was previously reported in adults ^16^. For treatment of unresectable IMTB, no clear recommendation has been defined, the role of chemotherapy is a topic of controversy, and anecdotal experience suggests that non-steroidal anti-inflammatory drugs (NSAIDs) may shrink large IMTBs to a more readily resectable size and configuration or eradicate them altogether. It is hypothesized that NSAIDs interfere with vascular endothelial growth factor (VEGF) signaling via inhibition of cyclooxygenase 2 (Cox-2). Target therapies possibly help to induce regression of neoplastic inflammatory masses via three different mechanisms: (1) suppression of the VEGF angiogenesis stimulation (Cox-2/prostaglandin/VEGF pathway), (2) direct inhibition of the proliferation of activated endothelial cells in which Cox-2 is known to be expressed, and (3) an anticytokine suppression effect on the inflammatory process ^16– 19^. There are other drug options, such as vinblastine and low-dose methotrexate ^18^. The armamentarium of systemic therapies has changed in our study period, and the role of classic chemotherapy should be discussed in light of the availability and efficacy of tyrosine kinase inhibitors for unresectable tumors or in case of predicted mutilating surgery.

### Leiomyoma

Bladder leiomyomas are rare, accounting for less than 1% of all mesenchymal bladder tumor subtypes, and are exceedingly rare in children. Clinical presentation includes urinary obstruction, frequent urination, dysuria, and hematuria ^20^. Characteristically, these lesions are a solitary, homogeneously attenuating mass. Excision is curative and carries no risk of recurrence or metastasis.

### Neuroendocrine tumors

***Paraganglioma.*** The origin of bladder paragangliomas is related to the embryonic rests of chromaffin cells in the sympathetic plexus of detrusor muscle. These tumors are exceedingly rare (<0.5% of all bladder tumors in adults and children combined).

Bladder paragangliomas are usually benign, but 10% may be malignant (often hormonally active). Clinical presentation might be related to catecholamine excess, including hypertension and headache, sometimes related to micturition ^21, 22^.

Paragangliomas tend to be vascular tumors with avid enhancement characteristics at MRI. A PET scan may be employed to confirm diagnosis and to identify metastatic lymph nodes. Pre-operative care—including adrenergic blockade—is necessary to avoid an intra-operative hypertensive crisis. Surgical resection is usually curative ^21, 22^.

***Neurofibroma.*** Up to 75% of bladder neurofibromas are detected in children, and the bladder is the most common genitourinary site for these tumors. Bladder wall spinal and autonomic nerves can be involved. Patients can be completely asymptomatic or present voiding dysfunction. Bladder tumors may be the first manifestation of neurofibromatosis type 1 (NF1), and the cutaneous stigmata of the syndrome is occasionally identified ^23, 24^.

Neurofibroma usually is depicted as a benign solid mass (focal lesion or diffuse bladder wall thickening). Homogeneous low signal intensity on T1-weighted images and high signal intensity on T2-weighted images are the most common patterns at MRI ^23, 24^. Differential diagnosis includes RMS and paraganglioma. Debulking resection provides symptom relief, as complete resection is difficult because of the plexiform nature of these neoplasms. Malignant transformation of neurofibromas in a malignant peripheral nerve sheath tumor is rare but should be considered in case of pain or a rapidly growing tumor or both.

The response to chemotherapy or radiotherapy is poor, and the majority of cases of NF1 with bladder dysfunction are managed by surgery. Surgery has inherent complications in these patients. Immunosuppression and chemotherapy increase the risk of infection, the need for extensive resection of these tumors leads to significant lymphedema, and skin invasion or malnutrition exposes these patients to poor healing. Because complete resection of genitourinary plexiform neurofibromas is often difficult, non-continent urinary diversions are the method of choice ^25^.

The best long-term outcome following malignant transformation is achieved after complete resection with clear margins. This means that, when malignization appears during follow-up, surgery must be proposed without delay ^26^.

### Vascular tumors

In children, vascular tumors are exceedingly rare within the bladder and usually manifest as painless recurrent macroscopic hematuria ^27, 28^. Sonographically, vascular tumors may present as a polypoid solid mass with increased vascularity or less commonly as a diffuse bladder wall thickening. Usually, these tumors are solitary and are seen at the bladder dome or the posterolateral bladder walls. Syndromes such as Klippel–Trénaunay–Weber, Sturge–Weber, and Proteus are associated with tumor multiplicity ^27, 28^.

Ablative treatment is the standard of care; a partial cystectomy may be necessary for large tumors. Sclerotherapy has emerged as an option of bladder hemangioma management following a successful experience with this method in other body sites.

Lymphatic and arteriovenous malformations and bladder wall telangiectasia (in the setting of ataxia–telangiectasia) are differential diagnosis. Epithelioid hemangioendothelioma and angiosarcoma should be considered in aggressive cases ^27, 28^.

### Urothelial neoplasms

Urothelial neoplasms in children are rare and predominantly non-invasive. Lesions were classified in accordance with the 2004 World Health Organization/International Society of Urological Pathology criteria as urothelial papillomas (UPs), PUNLMPs, and low-grade urothelial carcinomas and high-grade urothelial carcinomas.

At presentation, the most common symptom is painless hematuria. In the presence of hematuria, US must be performed. If a bladder lesion is identified, transurethral resection of the bladder should be performed. The lesions are usually solitary, non–muscle invasive, and of low grade (mainly UPs and PUNLMPs) ( Figure 2). There is no ideal follow-up protocol. Recurrence or progression is uncommon in patients younger than 20 years, the reported recurrence rate is 7%, and a single case of progression has been reported so far ^29, 30^.

***Papillary urothelial neoplasm of low malignant potentials.*** PUNLMPs are common bladder lesions in children and are used to describe a urothelial tumor that resembles exophytic urothelial. They are normally solitary and small (1–2 cm), commonly occur at the posterior lateral walls and ureteric orifices of the bladder, are non-invasive, and do not metastasize. About 35% of PUNLMPs reportedly recur after complete resection, and around 10% of them increase in size if they are not treated; therefore, regular imaging surveillance is advocated ^29, 30^.

***Urothelial papilloma.*** UPs are benign polypoid urothelial neoplasms that are infrequently reported in children. Microscopically, a broad vascular core covered by a normal urothelium and no cytologic atypia is the pattern.

UPs have a frond-like appearance in imaging. Transurethral excision is the treatment of choice. Because UPs seen in adults are known to recur, US follow-up has been advocated ^29, 30^.

***Urothelial carcinoma.*** Urothelial carcinomas are rare and, in children, have a different clinical course. Most of these tumors are solitary, have a low-grade morphology, are associated with a low risk of recurrence, and seldom involve the upper urinary tract. They are much more common in adolescents; only 30% of cases occur in children who are 10 years old or younger ^29, 30^.

Urothelial carcinoma may occur in childhood cancer–predisposing syndromes such as Costello syndrome and hereditary non-polyposis colorectal cancer syndrome. Augmented bladders have an increased risk of developing urothelial carcinoma, usually at bladder-intestinal anastomosis, and these tumors are frequently of high grade and have a more aggressive behavior ^27, 29, 30^.

No criteria have been established for treatment and follow-up for these children with urothelial carcinomas. For most patients, treatment is transurethral resection of the bladder tumor (TURBT). There is no recommendation of adjuvant therapy following resection as these tumors are rare in a pediatric age group. Some authors have performed partial cystectomy instead of TURBT in view of the risk of perforation of the pediatric bladder. Some reports also have given treatment with mitomycin C along with TURBT. There is no standard surveillance protocol for the imaging follow-up of patients who have received treatment, and recurrences have been reported, especially in cases with multiple tumors ^29– 31^.

### Nephrogenic adenoma

Nephrogenic adenoma (NA) of the urinary bladder is a metaplastic change in the urinary bladder and has papillary or cryptic structures similar to those seen in renal tubules. Microscopically, NA consists of small to medium tubules, cysts, or papillae coated by cuboid epithelium to low columnar epithelium with eosinophilic cytoplasm. Genitourinary trauma and chronic inflammation are factors that predispose patients to exfoliation of renal tubular cells, leading to sowing in the urinary tract, especially to the urinary bladder. Several patterns of growth (tubular, cystic, tubulocystic, papillary, and flat) have been reported in isolation or combined (mixed morphology) ^32^.

NAs, in contrast to other bladder tumors, commonly manifest with frequent urination rather than hematuria. For treatment, removal of the causative factors and excision of the lesion are required ^32^.

### Urachal adenocarcinoma

Urachal anomalies are thought to be associated with an increased risk of bladder adenocarcinoma in adults. Urachal anomalies are found incidentally in about 1% of children, and most anomalies are asymptomatic urachal remnants. The incidence of urachal malignancy in adults is extremely low (0.18 per 100,000 individuals yearly), and there is only one case described of urachal adenocarcinoma in children ^26^. It is therefore questionable that prophylactic resection of incidental urachal remnants plays a role in preventing future malignancies ^33^.
